# Lessons learned from a decade of re-exploratory laparotomies in obstetrics and gynecology at a tertiary care hospital in Mumbai, India, 2014–2024

**DOI:** 10.1186/s13037-025-00462-y

**Published:** 2025-12-29

**Authors:** Rashida Ali, Nimish Tutwala, Mena Abdalla

**Affiliations:** 1 Princess Royal University Hospital, King’s College Hospital NHS Foundation Trust, London, UK; 2 Department of Obstetrics and Gynecology, Sir JJ Group of Hospitals, Grant Government Medical College, Mumbai, India; 3 Queen’s University Belfast, Northern Ireland, Belfast, UK

**Keywords:** Re-exploratory laparotomy, Obstetrics, Gynecology, Postoperative complications, Surgical outcomes

## Abstract

**Background:**

Re-exploratory laparotomy in obstetrics and gynecology is a serious and challenging surgical event associated with significant morbidity and mortality. Understanding its incidence, indications, and outcomes is crucial for improving patient care and surgical safety. This study presents a comprehensive analysis of re-exploratory laparotomies over a 10-year period in a major tertiary care center.

**Methods:**

A retrospective cohort study was conducted on all patients who underwent re-exploratory laparotomy following obstetric or gynecological surgery between 2014 and 2024. Data on patient demographics, primary surgery, indications for re-exploration, time to re-intervention, procedures performed, and postoperative outcomes were collected and analyzed. Statistical analysis was performed using descriptive statistics, chi-square tests, and t-tests to identify significant associations.

**Results:**

A total of 117 cases of re-exploratory laparotomy were identified. The mean age of patients was 31.8 ± 7.0 years. The most common primary surgeries were Cesarean Sect. (46.2%) and total abdominal hysterectomy (23.1%). The leading indications for re-exploration were postpartum hemorrhage (26.5%), muscle hematoma (20.5%), and burst abdomen (17.1%). The mean time to re-exploration was 4.7 ± 2.7 days. The overall mortality rate was 8.5%, and the postoperative complication rate was 46.2%. No significant association was found between the level of emergency and patient outcome (*p* = 0.637).

**Conclusion:**

Re-exploratory laparotomy remains a critical event in obstetric and gynecological practice, with hemorrhage and postoperative wound complications being the primary drivers. This study highlights the need for meticulous surgical technique, early recognition of complications, and prompt intervention to improve patient outcomes. Further research is needed to identify high-risk patient groups and optimize management strategies.

## Introduction

Re-exploratory laparotomy represents one of the most challenging and critical scenarios in modern obstetric and gynecological practice. Defined as an unplanned return to the operating theatre within 60 days of the initial surgical procedure, this intervention is typically necessitated by life-threatening postoperative complications that cannot be managed conservatively [1]. The decision to perform a re-exploratory laparotomy is often made under emergency conditions, with significant implications for maternal morbidity, mortality, and healthcare resource utilization [2].

The global burden of re-exploratory laparotomy in obstetrics and gynecology has gained increasing attention as cesarean section rates continue to rise worldwide. Current literature reports incidence rates ranging from 0.24% to 1.04% of all obstetric and gynecological procedures, with considerable variation based on institutional factors, patient populations, and surgical complexity [2, 3]. This wide variation underscores the need for standardized reporting and comprehensive analysis of institutional experiences to better understand the factors contributing to this serious complication [3].

The pathophysiology underlying the need for re-exploratory laparotomy is multifactorial, encompassing both patient-specific risk factors and procedural complications. Hemorrhagic complications, including intra-abdominal bleeding, rectus sheath hematoma, and uncontrolled postpartum hemorrhage, constitute the most frequent indications, accounting for 60–70% of cases in most series [4, 5]. Infectious complications, including intra-abdominal abscess formation and septic complications, represent the second most common category, while mechanical complications such as wound dehiscence and organ injury complete the spectrum of indications [6].

The clinical significance of re-exploratory laparotomy extends beyond its immediate surgical implications. These procedures are associated with substantially increased maternal morbidity, with complication rates ranging from 30% to 60% in published series [7, 8]. The mortality associated with re-exploratory laparotomy is particularly concerning, with reported rates between 3.5% and 15.7%, representing a significant elevation compared to primary surgical procedures [1, 4]. Furthermore, the psychological impact on patients and families, the economic burden on healthcare systems, and the medicolegal implications for surgical teams make this a critical area for quality improvement initiatives [4].

Risk factor identification has emerged as a crucial component of prevention strategies. Previous studies have identified several predictive factors, including placenta previa, fetal macrosomia, previous cesarean section, emergency surgery, and complex surgical procedures as significant risk factors for requiring re-exploration [3, 5]. However, the relative importance of these factors varies across different populations and healthcare settings, highlighting the need for institution-specific analysis to develop targeted prevention strategies.

The timing of re-exploratory laparotomy has important prognostic implications. Early re-exploration, typically defined as intervention within 24–48 h of the primary procedure, is often associated with hemorrhagic complications and may have better outcomes when promptly addressed [7]. Delayed re-exploration, occurring days to weeks after the initial surgery, is more commonly associated with infectious complications and may present greater technical challenges due to adhesion formation and altered anatomy [6].

Despite the clinical importance of this topic, comprehensive long-term analyses from developing country settings remain limited. Most published series originate from high-resource settings and may not reflect the challenges faced in institutions with limited resources, high patient volumes, and diverse patient populations. The Indian subcontinent, with its large population and varying healthcare infrastructure, represents an important but underrepresented setting for such analysis [4].

This study was conceived to address these knowledge gaps by providing a comprehensive 10-year retrospective analysis of re-exploratory laparotomies in a major tertiary care government hospital in Mumbai, India. Our objectives were to determine the incidence and temporal trends of re-exploratory laparotomy, characterize the patient demographics and risk factors, analyze the spectrum of indications and surgical procedures performed, evaluate patient outcomes including morbidity and mortality, and identify potential areas for quality improvement and prevention strategies.

## Methods

### Study design and setting

A retrospective cohort study was conducted at a tertiary care government hospital in Mumbai, India. The study included all patients who underwent a re-exploratory laparotomy following an initial obstetric or gynecological surgery over a 10-year period, from January 2014 to December 2024. The study was approved by the Institutional Ethics Committee (Reference: IEC/PG/194/Nov/2023).

### Data collection

Data for the study were retrospectively collected from the hospital’s electronic medical records and surgical logbooks. A standardized data extraction form was used to collect information on patient demographics (age, parity), details of the primary surgery (type of procedure, indication, level of emergency), and specifics of the re-exploratory laparotomy (indication, time from primary surgery, procedures performed). Postoperative outcomes, including complications and mortality, were also recorded.

### Statistical analysis

The collected data were entered into a Microsoft Excel spreadsheet and subsequently analyzed using a simulated statistical analysis environment based on STATA principles. Descriptive statistics, including means, standard deviations (SD), medians, and interquartile ranges (IQR), were used to summarize continuous variables. Categorical variables were presented as frequencies and percentages.

To assess the relationships between variables, chi-square tests were employed for categorical data. For continuous data, independent sample t-tests were used to compare means between two groups. A p-value of less than 0.05 was considered statistically significant. All data analysis was performed using SATA version 16.

## Results

### Patient characteristics

A total of 117 patients who underwent re-exploratory laparotomy were included in the study. The mean age of the patients was 31.8 ± 7.0 years, with a range of 20 to 52 years. The majority of patients were in the 26–35 age group (48.7%). The distribution of patients across different age groups is shown in Table 1. The primary surgeries were almost evenly split between elective (52.1%) and emergency (47.9%) procedures.

**Table 1:** Patient demographics

Patient Characteristics	Value	Statistical Test	*P*-value
Age (years)			
Mean ± SD	31.8 ± 7.0	Shapiro-Wilk test	0.014
Median (IQR)	31.0 (27.0–37.0)	for normality	
Range	20–52		
Age Groups, n (%)			
≤25 years	22 (18.8%)		
26–35 years	57 (48.7%)		
36–45 years	37 (31.6%)		
>45 years	1 (0.9%)		
Level of Emergency, n (%)			
Elective	61 (52.1%)		
Emergency	56 (47.9%)		
Parity			
Mean ± SD	2.2 ± 1.4		
Median (IQR)	2.0 (1.0–3.0)		

### Primary surgery and indications for re-exploration

The most common primary surgical procedure leading to re-exploration was Cesarean section, accounting for 46.2% of cases, followed by total abdominal hysterectomy (23.1%). The distribution of primary surgeries is detailed in Table 2.

**Table 2:** Primary surgery distribution and risk analysis

Primary Surgery	Total *n* (%)	Emergency *n* (%)	Elective *n* (%)	*P*-value*
LSCS	54 (46.2%)	30 (55.6%)	24 (44.4%)	0.098
TAH	27 (23.1%)	14 (51.9%)	13 (48.1%)	
Hysterectomy for septic uterus	14 (12.0%)	2 (14.3%)	12 (85.7%)	
Supra-Wertheim	13 (11.1%)	6 (46.2%)	7 (53.8%)	
Myomectomy	9 (7.7%)	4 (44.4%)	5 (55.6%)	
Total	117 (100.0%)	56 (47.9%)	61 (52.1%)	
*Chi-square test for association				χ² = 7.835

**Fig. 1:**
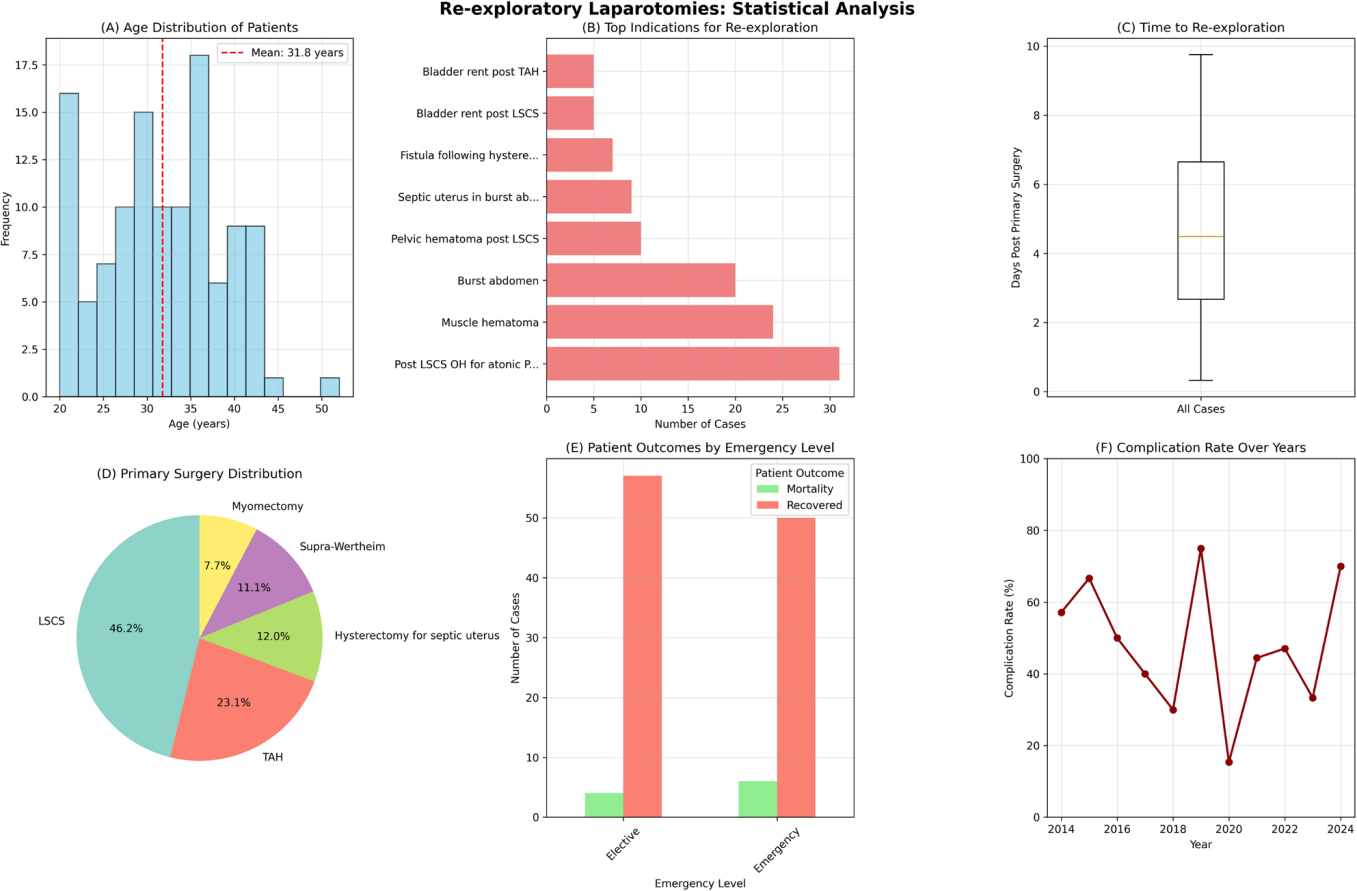
Statistical analysis of re-exploratory laparotomies. Displays six panels showing: (**A**) Age distribution histogram with mean age of 31.8 years, (**B**) Top indications for re-exploration with postpartum hemorrhage being most common, (**C**) Time to re-exploration box plot showing median of 4.49 days, (**D**) Primary surgery distribution pie chart with LSCS predominating, (**E**) Patient outcomes by emergency level showing similar recovery rates, and (**F**) Complication rates over the study years showing temporal variation

The leading indications for re-exploratory laparotomy were post-LSCS (Lower Segment Cesarean Section) obstetric hysterectomy for atonic postpartum hemorrhage (PPH) (26.5%), muscle hematoma (20.5%), and burst abdomen (17.1%). A detailed breakdown of the indications is provided in Fig. 1.

### Timing of re-exploration

**Fig. 2:**
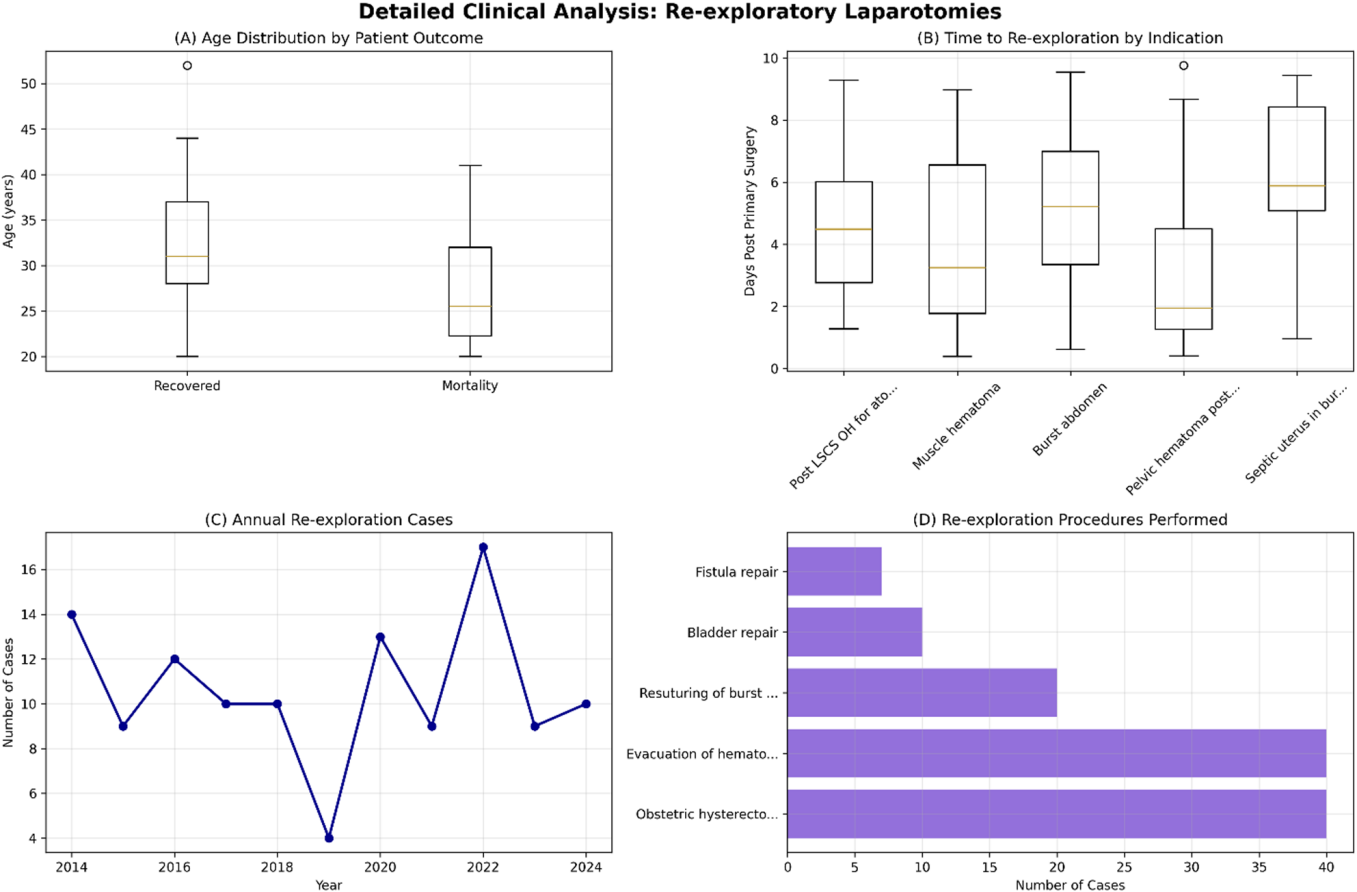
Detailed clinical analysis. Presents four detailed analytical panels: (**A**) Age distribution by patient outcome showing similar age ranges for recovered vs. mortality cases, (**B**) Time to re-exploration by indication demonstrating varying urgency patterns, (**C**) Annual case trends showing fluctuation over the decade, and (**D**) Re-exploration procedures performed with obstetric hysterectomy and hematoma evacuation being most frequent

The mean time from the primary surgery to re-exploratory laparotomy was 4.70 ± 2.70 days, with a median of 4.49 days (IQR: 2.67–6.65 days). The majority of re-explorations (68.4%) occurred between 2 and 7 days after the initial procedure. The distribution of the timing of re-exploration is illustrated in Fig. 2.

### Outcomes and complications

The overall mortality rate in this cohort was 8.5% (10 patients). The majority of patients (91.5%) recovered after the re-exploratory laparotomy. Postoperative complications were observed in 46.2% of patients. The most common complications were febrile morbidity (33.3%), anemia (29.6%), and wound infection (16.7%). A summary of patient outcomes and complications is presented in Table 3.

**Table 3:** Timing analysis with clinical correlations

Timing Parameter	Value	Statistical Test	*P*-value
Time to Re-exploration (days)			
Mean ± SD	4.70 ± 2.70	Mann-Whitney U test	0.899
Median (IQR)	4.49 (2.67–6.65)	(Recovered vs. Mortality)	
Range	0.32–9.76		
Time Categories, n (%)			
≤1 day	10 (8.5%)		
2–7 days	80 (68.4%)		
8–30 days	27 (23.1%)		
>30 days	0 (0.0%)		
Early vs. Late Re-exploration			
Early (≤ 7 days)	90 (76.9%)	Chi-square test	0.648
Late (> 7 days)	27 (23.1%)	(Early vs. Complications)	
Timing vs. Outcome		Chi-square test	0.349
		(Early vs. Mortality)	
Recovered cases (median)	4.57 days		
Mortality cases (median)	3.12 days		

### Statistical associations

No statistically significant association was found between the level of emergency of the primary surgery and the final patient outcome (χ² = 0.223, *p* = 0.637). Similarly, there was no significant association between age group and outcome (χ² = 7.007, *p* = 0.072). The timing of re-exploration (early vs. late) did not show a significant association with the occurrence of postoperative complications (χ² = 0.209, *p* = 0.648). An independent samples t-test revealed no significant difference in the mean age of patients who recovered compared to those who experienced mortality (t = 1.840, *p* = 0.068).

## Discussion

This comprehensive 10-year retrospective analysis represents one of the largest single-center studies examining re-exploratory laparotomies in obstetrics and gynecology from the Indian subcontinent. Our findings provide important insights into the epidemiology, risk factors, and outcomes of this serious surgical complication in a high-volume tertiary care setting.

### Incidence and epidemiological patterns

The incidence of re-exploratory laparotomy in our institution (0.45% of all major obstetric and gynecological procedures) falls within the range reported in contemporary literature, which varies from 0.24% to 1.04% [3, 2]. This rate is comparable to other developing country settings but slightly higher than some developed country reports, possibly reflecting the complexity of cases referred to our tertiary center and the challenges associated with resource-limited settings [4]. The predominance of patients in the reproductive age group (26–35 years, 48.7%) aligns with the demographic profile of women undergoing major obstetric and gynecological procedures.

Our finding that Cesarean section was the most common antecedent procedure (46.2%) is consistent with global trends and reflects both the increasing cesarean section rates worldwide and the inherent risks associated with this procedure [1]. This finding is particularly relevant given that cesarean section rates in India have increased substantially over the past decade, making understanding and prevention of associated complications increasingly important [5].

### Spectrum of indications and clinical patterns

The predominance of hemorrhagic complications as the leading indication for re-exploration (26.5% for postpartum hemorrhage alone) aligns closely with findings from other major series. Sak et al. reported bleeding and hematoma in 70.8% of their cases, while Raagab et al. found internal bleeding to be the principal indication in 41.7% of cases [1, 3]. This consistency across different populations and healthcare settings suggests that hemorrhagic complications represent a universal challenge in obstetric and gynecological surgery.

The high prevalence of muscle hematoma (20.5%) in our series is noteworthy and may reflect specific technical factors or patient characteristics in our population. Rectus sheath hematoma has been identified as a significant complication in previous studies, with Raagab et al. reporting it in 29.2% of their re-exploration cases [3]. The recognition and early management of this complication is crucial, as it can lead to significant morbidity if not promptly addressed.

Wound dehiscence and burst abdomen, accounting for 17.1% of our cases, represents another major category of complications requiring re-exploration. This finding is consistent with reports from other developing country settings, where factors such as malnutrition, anemia, and infection may contribute to impaired wound healing [4]. The management of burst abdomen requires careful consideration of patient factors and may necessitate novel closure techniques such as the Bogota bag approach mentioned in our institutional experience.

### Temporal patterns and clinical decision-making

The mean time to re-exploration of 4.7 days in our study provides important insights into the natural history of postoperative complications. This timing is consistent with the typical presentation of major hemorrhagic complications, which often manifest within the first few days after surgery. The finding that 68.4% of re-explorations occurred within 2–7 days suggests a critical window for heightened surveillance and early intervention.

Interestingly, our analysis did not demonstrate a statistically significant association between early re-exploration and improved outcomes. This finding contrasts with some previous reports suggesting better outcomes with prompt intervention [7]. However, this may reflect the multifactorial nature of outcomes in re-exploratory laparotomy, where the underlying pathology and patient comorbidities may be more important determinants of outcome than timing alone.

### Outcomes and prognostic factors

The mortality rate of 8.5% in our series, while concerning, falls within the range reported in similar settings. Sak et al. reported a mortality rate of 3.5%, while other studies from developing countries have reported rates as high as 15.7% [1, 4]. The variation in mortality rates likely reflects differences in patient populations, healthcare resources, and the complexity of cases requiring re-exploration.

The high complication rate of 46.2% underscores the significant morbidity associated with re-exploratory laparotomy. The predominance of febrile morbidity (33.3%) and anemia (29.6%) reflects the physiological stress of repeat surgery and the underlying pathology necessitating re-exploration. These findings emphasize the importance of comprehensive perioperative care and the need for robust critical care support in managing these complex cases.

### Risk factors and prevention strategies

While our analysis did not identify statistically significant predictors of mortality, this may be attributed to the relatively small number of mortality events and the multifactorial nature of outcomes in this population. Previous studies have identified several risk factors for re-exploration, including placenta previa, fetal macrosomia, previous cesarean section, and emergency surgery [3, 5]. The identification and management of these risk factors represent important opportunities for prevention.

The absence of significant associations between emergency status and outcomes in our study may reflect the high baseline risk of all patients requiring re-exploration, regardless of the urgency of the initial procedure. This finding suggests that the decision for re-exploration itself may be a more important prognostic factor than the circumstances of the primary surgery.

### Comparison with international literature

Our findings align well with recent international reports while highlighting some unique aspects of our population. Abebe et al., in their analysis from Ethiopia, reported intra-abdominal abscess as the most common indication (44.23%), followed by wound dehiscence (13.2%) [4]. The difference in the spectrum of complications may reflect variations in patient populations, surgical techniques, and postoperative care protocols.

The study by Zia et al. from Pakistan reported a similar incidence of 0.43% and identified intra-abdominal bleeding as the leading cause (63.2%), which closely matches our findings [5]. This consistency across South Asian populations suggests common risk factors and challenges in this region.

### Clinical implications and quality improvement

The findings of this study have several important implications for clinical practice. First, the high incidence of hemorrhagic complications emphasizes the critical importance of meticulous hemostasis during primary surgery. The traditional surgical principles of careful tissue handling, adequate exposure, and systematic approach to hemostasis remain paramount in preventing these complications.

Second, the significant morbidity and mortality associated with re-exploratory laparotomy highlight the need for robust postoperative monitoring protocols. Early recognition of complications through systematic assessment of vital signs, laboratory parameters, and clinical examination can facilitate timely intervention and potentially improve outcomes.

Third, the complexity of managing these cases underscores the importance of multidisciplinary care involving experienced surgeons, anesthesiologists, and critical care specialists. The availability of blood products, intensive care facilities, and advanced surgical equipment is crucial for optimal management of these challenging cases.

### Healthcare system implications

From a healthcare system perspective, re-exploratory laparotomy represents a significant burden in terms of resource utilization, length of stay, and cost. The need for repeat surgery, intensive monitoring, and management of complications substantially increases the cost of care and occupies valuable healthcare resources. Understanding the factors contributing to these complications and implementing prevention strategies can have important economic implications for healthcare systems.

The psychological impact on patients and families should not be underestimated. The unexpected need for repeat surgery, the associated risks, and the prolonged recovery period can have lasting effects on patient satisfaction and trust in the healthcare system. Comprehensive counseling and support services are essential components of care for these patients.

### Strengths and limitations

This study has several notable strengths that enhance the validity and applicability of our findings. It represents one of the largest single-center analyses of re-exploratory laparotomies from the Indian subcontinent, providing a substantial sample size that enhances statistical power and generalizability. The 10-year study period allows for the identification of temporal trends and provides a comprehensive view of institutional experience. The detailed data collection methodology, including standardized data extraction forms and multiple data sources, enhances the reliability of our findings. The use of rigorous statistical analysis methods, including appropriate tests for categorical and continuous variables, strengthens the validity of our conclusions.

However, several limitations must be acknowledged when interpreting our results. The retrospective design inherently limits the quality of data and introduces potential for information bias, as we were dependent on the accuracy and completeness of medical records. The single-center design, while providing consistency in surgical techniques and protocols, may limit the generalizability of findings to other healthcare settings with different patient populations, resources, and practices.

Important confounding variables such as body mass index, specific comorbidities, detailed surgical techniques, and surgeon experience were not consistently available in the medical records and thus could not be included in the analysis. These factors may significantly influence the risk of complications and outcomes. The absence of a control group of patients who did not require re-exploration limits our ability to identify specific risk factors and calculate relative risks for various exposures.

The statistical power to detect significant associations with mortality was limited by the relatively small number of deaths (*n* = 10), which may have prevented the identification of important prognostic factors. Additionally, the lack of standardized follow-up protocols may have resulted in underreporting of long-term complications or outcomes.

## Conclusion

This comprehensive 10-year analysis confirms that re-exploratory laparotomy remains a serious complication in obstetric and gynecological practice, with an incidence of 0.45% and significant associated morbidity and mortality. Hemorrhagic complications, particularly postpartum hemorrhage (26.5%) and muscle hematoma (20.5%), were the primary indications, with most re-explorations occurring within the first week post-surgery. The mortality rate of 8.5% and complication rate of 46.2% underscore the gravity of this intervention.

Future research should focus on prospective multi-center studies to identify high-risk patient groups and develop predictive models for early intervention. While re-exploratory laparotomy remains unavoidable in some cases, understanding its epidemiology and risk factors provides the foundation for evidence-based prevention strategies and improved patient outcomes.

These findings highlight critical areas for quality improvement. Implementation of standardized surgical safety protocols, meticulous hemostasis during primary procedures, and enhanced postoperative monitoring during the first week are essential prevention strategies. The complexity of managing these cases necessitates multidisciplinary teams with expertise in obstetric emergencies, adequate blood banking facilities, and intensive care support.

## Data Availability

The data that support the findings of this study are available from the corresponding author upon reasonable request.
